# Mapping Proteome and Lipidome Changes in Early-Onset Non-Alcoholic Fatty Liver Disease Using Hepatic 3D Spheroids

**DOI:** 10.3390/cells11203216

**Published:** 2022-10-13

**Authors:** Helle Sedighi Frandsen, Joel Mario Vej-Nielsen, Lauren Elizabeth Smith, Lang Sun, Karoline Lindgaard Mikkelsen, Annemette Præstegaard Thulesen, Christina Erika Hagensen, Fuquan Yang, Adelina Rogowska-Wrzesinska

**Affiliations:** 1 Department of Biochemistry and Molecular Biology, University of Southern Denmark, 5230 Odense M, Denmark; 2 Sino-Danish College (SDC), University of Chinese Academy of Sciences, 380 Huaibeizhuang, Beijing 100101, China; 3 Key Laboratory of Protein and Peptide Pharmaceuticals & Laboratory of Proteomics, Institute of Biophysics, Chinese Academy of Sciences, Beijing 100101, China; 4 University of Chinese Academy of Sciences, Beijing 101408, China; 5 CelVivo ApS, 5491 Blommenslyst, Denmark

**Keywords:** 3-dimensional cell culture, spheroids, human hepatocytes, fatty liver disease, NAFLD, steatosis, lipidomics, proteomics

## Abstract

Non-alcoholic fatty liver disease affects one-fourth of the world’s population. Central to the disease progression is lipid accumulation in the liver, followed by inflammation, fibrosis and cirrhosis. The underlying mechanism behind the early stages of the disease is poorly understood. We have exposed human hepatic HepG2/C3A cells-based spheroids to 65 μM oleic acid and 45 μM palmitic acid and employed proteomics and lipidomics analysis to investigate their effect on hepatocytes. The treatment successfully induced in vivo hallmarks of NAFLD, as evidenced by intracellular lipid accumulation and increased ATP levels. Quantitative lipidome analysis revealed an increase in ceramides, LPC and saturated triglycerides and a decrease in the ratio of PC/PE, similar to the changes observed in patients’ liver biopsies. The proteomics analysis combined with qPCR showed increased epithelial to mesenchymal transition (EMT) signalling. Activation of EMT was further validated by transcriptomics in TGF-β treated spheroids, where an increase in mesenchymal cell markers (N-cadherin and collagen expression) was found. Our study demonstrates that this model system thus closely echoes several of the clinical features of non-alcoholic fatty liver disease and can be used to investigate the underlying molecular changes occurring in the condition.

## 1. Introduction

A sedentary lifestyle with high caloric intake can lead to an excess accumulation of lipids in the liver, known as non-alcoholic fatty liver disease (NAFLD) [1]. NAFLD is a spectrum of conditions, ranging from simple steatosis to non-alcoholic steatohepatitis, fibrosis, possibly cirrhosis, and hepatocellular carcinoma [2]. The mechanism underlying the onset of the disease is poorly understood [3]. This lack of knowledge is why no FDA-approved drug is available for NAFLD [4].

Diagnosis of NAFLD relies on abnormal blood test results like elevated alanine transaminase (ALT) and aspartate transaminase (AST), imaging studies identifying fatty infiltrate in the liver or histopathological analysis of liver biopsies [5]. However, blood tests do not always pick up NAFLD, and the disease can remain asymptotic until the late stages. That contributes to the lack of biomaterial to investigate how NAFLD progresses and our poor understanding of its mechanisms [6,7,8].

A wide range of conditions can increase the risk of NAFLD. The most important are obesity, insulin resistance, hyperglycaemia, and high levels of fats, particularly triglycerides, in the blood [2,9]. It is still unclear why some people accumulate fat in the liver while others do not. High-fat diet-associated obesity is widespread in patients with NAFLD and is emerging as one of the universal causes of liver disease worldwide [10].

3D cell culture is a promising system for investigating NAFLD. It promotes a microenvironment similar to that found in vivo in the human body ensuing in vitro models with a higher predictive power [11]. Such an approach allows for studying NAFLD in its early stages in human cells, avoiding pitfalls associated with animal models [12] or monolayer cell cultures [13].

Omics provides a large-scale approach to studying holistic changes during the progression of NAFLD [14]. Genomics, for example, has been heavily utilized in analysing and finding polymorphisms in NAFLD patients [15]. Other omics strategies have also been employed to a lesser extent since they require scarce liver biopsies as sample material. Proteomics studies assist in understanding the mechanisms of pathology by analysing the protein expression patterns that reflect alterations that occur after genes’ expression. Lipidomics is a newer omics technology that shows great promise for NAFLD research. Information about the specific lipid classes accumulated has indicated lipid’s essential role in disease progression [16]. This study provides the first-ever analysis of both the proteome and lipidome of a 3D liver model with induced steatosis.

This study aimed to investigate the molecular alterations in hepatocytes exposed to elevated levels of fatty acids. We have used an in vitro model of the healthy adult liver using HepG2/C3A cell-based spheroids [17,18]. To mimic the key NAFLD risk factor, increased fat intake, we added 45 μM palmitic acid (PA) and 65 μM oleic acid (OA) to spheroid cell culture media. These concentrations correspond to the increase in PA and OA observed in the plasma of NAFLD patients compared to healthy individuals [19], correlate with pathophysiological conditions, and are significantly lower than in other previously published studies [20,21,22].

We show that the model recapitulates the key molecular features of NAFLD, including lipid accumulation and other changes in the lipidome such as those observed in vivo. We demonstrate increased TGF-β1 signalling and the development of a mesenchymal-like phenotype of hepatocytes.

## 2. Materials and Methods

### 2.1. Monolayer Cell Culture

The immortal human hepatocyte cell line, HEPG2/C3A (ATCC CRL-10741), from here on termed C3A, was cultured at 37 °C, in 5% CO_2_ and 95% air, and in 87.5% Dulbecco’s Modified Eagle’s Medium (containing 5.6 mM glucose) supplemented with 1% non-essential amino acids, 10% foetal bovine serum (FBS), 0.5% penicillin/ streptomycin, and 1% GlutaMAX. Media was changed every 2–3 days, and the cells were sub-cultured at a confluence of 60–90% three times before initiation of any experiment [17].

### 2.2. Formation and Maintenance of Spheroids

Spheroids were generated using a previously established protocol [23]. Briefly, AggreWell400 plates (Stemcell Technologies, Vancouver, Canada) were washed with Rinsing Solution (Stemcell Technologies), prefilled with 0.5 mL growth medium per well, and seeded with a single-cell suspension containing 1.2 million cells per well to yield 1000 cells per/microwell. The plate was centrifuged for 3 min at 130 RCF and incubated at 37 °C for 24 h to allow aggregation. The spheroids were transferred to 10 mL rotary bioreactors (CelVivo ApS, Blommenslyst, Denmark). The bioreactors were filled with growth media and placed in an incubator (37 °C, 5% CO_2_, 95% air, humidified) on a Bio Array Matrix (BAM) rotary unit (CelVivo ApS, Blommenslyst, Denmark). The rotation speed was adjusted according to the increasing size of spheroids during culture. Growth medium was exchanged a week thrice (90% of the volume). Three hundred spheroids were cultured per bioreactor from day 7, with five bioreactors in total, until day 19, where the bioreactors were pooled and divided into nine bioreactors containing 85 spheroids each.

### 2.3. Spheroids Treatment (Palmitic and Oleic Acid or TGF-β1)

Spheroids were cultured 21 days before treatment with 65 μM oleic acid (OA), and 45 μM palmitic acid (PA) suspended in growth media. The OA and PA 100× stock solutions were prepared in 1% fatty acid-free bovine serum albumin [24]. Spheroids and culture media samples were taken after 0, 1, 2, 4, and 7 days of fatty acid exposure. A separate TGF-β1 exposure experiment (2 ng/mL) was initiated after eight days of culture and continued for 14 days. The spheroids thus were exposed to fresh TGF-β1 at each media change three times a week. Samples were collected for qPCR after 7 and 14 days of treatment.

### 2.4. Monitoring Spheroid Size

Phase-contrast light microscopy images of intact spheroids were used to determine spheroid size. Images were captured using Olympus IX81 motorized microscope and Olympus DP71 camera. The planar area of spheroids was measured using AnalySiS Docu software (Soft Imaging System, Berlin, Germany).

### 2.5. ATP Assay

Spheroids were transferred to 96-well plates for ATP measurements using CellTiter-Glo luminescent cell viability assay (Promega, Nacka, Sweden). One spheroid per well was used, and five spheroids were used per condition (*n* = 5). The measurements were performed following the manufacturer’s recommendations and as previously described [23]. Spheroids were disrupted by repeated pipetting. The results were recorded using the FLUOstar OPTIMA plate reader (BMG Labtech, Ortenberg, Germany).

### 2.6. Quantification of Intracellular Lipid Content

AdipoRed Adipogenesis Assay Reagent (Lonza, Walkersville, MD, USA) was used to quantify the levels of intracellular lipids accumulated in spheroids as described before [25]. Each spheroid was placed in a single well of a black 96-well plate (*n* = 5). Growth media was removed, and 200 μL staining solution (2.5% AdipoRed Adipogenesis Assay Reagent) was added to each well. Spheroids were disrupted manually by pipetting, incubated for 10 min, and measured on a FLUOstar OPTIMA plate reader.

### 2.7. Glucose Consumption and Glycogen Storage

After four days of fatty acid exposure, glucose levels in media samples were tested at 0, 4, 8, 24, 48 and 72 h after media change. As previously described, glucose content was measured immediately after sampling using a Onetouch Vita blood glucose meter (Mediq Danmark, Brøndby, Denmark) [23]. Total glycogen was measured in spheroids (*n* = 3) using a Glycogen assay kit (Sigma Aldrich, Saint Louis, MO, USA), as previously described [18]. Samples were sonicated, followed by assay measurement performed according to the manufacturer’s manual using a FLUOstar OPTIMA plate reader.

### 2.8. Fluorescent Microscopy

Spheroids were fixed for one hour in 4% paraformaldehyde in PBS at room temperature (21 °C) and embedded in Tissue Tek O.C.T. Compound (Sakura Finetek USA, Inc., Torrance, CA, USA) in a 1.5-millilitre Eppendorf tube (3 spheroids per tube) and snap-frozen [23]. Samples were cut into 30-micrometre slices, attached to super frost plus slides and stored at −80 °C until use. Slices were permeabilized using 0.3% TritonX-100 in PBS for 10 min, stained using phalloidin (1:100) (Molecular Probes, Eugene, OR, USA) and Nile red (1:100) (Sigma-Aldrich) and DAPI (1:1000) (Invitrogen, Waltham, MA, USA) for one hour and washed thrice in PBS. Images were captured using NIKON NIS-elements AR software and an A1-R-HD25 confocal microscope. Images were captured at 100× magnification and deconvoluted using the Richardson–Lucy deconvolution algorithm. 

### 2.9. Data Analysis of Functional Assays

Statistical analysis for planimetry, ATP, glycogen, glucose consumption and lipid accumulation was carried out using GraphPad Prism 9. The data were analysed using Student’s *t*-test with the Holm–Sidak post hoc test. A statistical difference was defined by a *p*-value equal to or below 0.05.

### 2.10. Gene Expression Analysis

Ten spheroids from each condition were sampled in triplicate, washed thrice in PBS, transferred to 500 μL TRIzol reagent (Invitrogen), disintegrated using a pipette, and stored at −80 °C. Samples were thawed on ice, and 5 μL of 2-mercaptoethanol and 100 μL chloroform were mixed for 30 s, followed by 15 min incubation on ice and 10 min centrifugation at 4 °C. The upper phase was transferred to a new tube, and 100 μL of 70% ethanol was added. According to the supplier’s manual, RNA was extracted using RNeasy Minikit (Qiagen, Hilden, Germany). RNA concentration was measured using IMPLEN Nanophotometer N60 (Fisher Scientific, Hampton, NY, USA). Gene expression was investigated as described by Stampar et al. [26]. cDNA production was performed using the cDNA High-Capacity Archive Kit (Applied Biosystems), following the manufacturer’s manual, using one μg of RNA on a PCRmax Alpha Thermal Cycler (Fisher Scientific). cDNA was used for further analysis using Taqman gene expression assays of 6 genes (60 ng RNA, (ACTA2: Hs00426835_g1, ALB: HS00910225_m1, TGFB1:Hs00998133_m1, CDH1: Hs01023895_m1, CDH2: Hs00983056_m1)) and COL1A1 (125 ng RNA, (COL1A1: Hs00164004_m1)) (Applied Biosystems, Waltham, MA, USA). The assays were performed according to the manufacturer’s manual in a 384 well plate format with a total volume of 10 μL in each well in technical duplicates and biological triplicates. Analysis was performed using Taqman Universal PCR master mix (Applied Biosystems) and a Lightcycler 480 (Roche). The crossing point (Cp) (second derivative method) of qPCR runs was determined for each well by Lightcycler^®^ 480 SW 1.5.1 software. Cp was further used for calculating relative gene expression. Data were normalized to the total RNA analysed [27].

### 2.11. Lipidomics

Lipids were extracted by a modified version of Lovric’s methyl-tert-butyl ether (MTBE) method [28]. Five spheroids per sample were disrupted in 100 μL PBS by sonication. Protein determination was made using the BCA protein assay kit (Thermo Scientific, Waltham, MA, USA), and the equivalent of 170 μg of protein was transferred to a new tube. Lipid internal standards containing ceramides (Cer), lysophosphatidylcholine (LPC), lysophosphatidylethanolamine (LPE), phosphatidylcholine (PC), phosphoethanolamine (PE), phosphoglycerol (PG) and phosphatidylserine (PS)) were added to each sample at a final concentration of 4 μg/mL and 12 μg/mL of diacylglycerol (DG) and triacylglycerol (TG) (Sigma-Aldrich, Saint Louis, MO, USA). A detailed list of internal standards can be found in Supplementary File S1. Subsequently, 300 μL of methanol was added to the samples and mixed for 10 min, followed by 500 μL MTBE (10 min mixing). After adding 250 μL of water and 10 min of centrifugation, the upper organic layer was collected. The extraction was performed twice, and the organic layer was lyophilized and resuspended in chloroform/methanol (1:1, 10 mM ammonium acetate). Each sample (2 μL) was separated using an Acquity UPLC HSS T3 column (Waters, 2.1 × 100 mm, 1.8 μm) coupled to a Dionex UltiMate 3000 nLC system (Thermo Fisher Scientific) with a flow rate of 250 μL/min. The gradient ranged from 0% solvent B (methanol/isopropyl alcohol 1:4, 5 mM ammonium acetate) at 0–1 min, 1–6 min 60% solvent B, 6–18 min 100% solvent B and 18–20 min 100% solvent B. Solvent A was (methanol/acetonitrile/water (1:1:1), 5 mM ammonium acetate)). Data acquisition was performed in data-dependent acquisition mode by a Triple TOF 5600 mass spectrometer (AB Sciex, Framingham, MA, USA) coupled to electrospray ionization (ESI) source in positive ion mode. See Supplementary File S1 for additional mass spectrometry settings. Data analysis was performed using Lipid Data Analyzer software developed by Hartler et al. [29]. Absolute values were calculated using internal standards. Lipid species were filtered using Perseus, and only species present in at least two replicas of each condition were used for further analysis [30]. Lipid species recognized by the compound name database in Metaboanalyst 5.0 (combining HMDB, PubChem and LIPID MAPS databases) were further used for statistical analysis in Metaboanalyst [31]. Metabolomics data have been deposited to the EMBL-EBI MetaboLights database [32] with the identifier MTBLS3580. The complete dataset is deposited at https://www.ebi.ac.uk/metabolights/ under accession number MTBLS3580. A list of identified lipids can be found in Supplementary Tables S1 and S2.

### 2.12. Proteomics

Five spheroids per condition were pooled for the isolation of proteins. Spheroids were disrupted mechanically with vigorous pipetting; the cells were lysed (50 mM TEAB, 1% SDC, 10 mM TCEP, 40 mM chloroacetamide, protease inhibitors and phosphatase inhibitors) following a published protocol [33]. Cell lysates were heated to 80 °C for 10 min, mixed and sonicated on ice. The protein concentration of samples was determined using tryptophan fluorescence [34], and 100 μg protein was loaded onto a spin filter (10 KDa) (Sartorius, Göttingen, Germany) for filter-assisted sample preparation-based digestion [33]. Before LC-MSMS analysis, peptides were resuspended in 2% acetonitrile (ACN)/0.1% trifluoroacetic acid and an aliquot was taken for amino acid quantitation [35]. One μg of the peptide mixture of each sample was utilized for proteomics analysis. Peptides were captured on a pre-column (Acclaim PepMap 100 C18, Thermo Fisher Scientific) and separated using an in-house packed column (120 Å pore size, C18) using the FlashPack method [36]. The column was heated to 50 °C, and the gradient was 8–35% solution B (99.99% ACN, 0.01% formic acid) over 160 min at 550 nL/min using a Dionex UltiMate 3000 nanoLC system (Thermo Fisher Scientific). Solution A was 0.1% formic acid in water. Peptides, separated online using reversed-phase liquid chromatography, were analysed by MS/MS using an Orbitrap Fusion Lumos Tribrid mass spectrometer (Thermo Fisher Scientific). Additional mass spectrometry settings can be found in Supplementary File S1. Initial data analysis was performed with MaxQuant (v 1.6.2.10) [37], using the Andromeda search engine [38] against the human UniProt Reference Proteome database (containing Swiss-Prot proteins including isoforms) (downloaded 25 February 2019, including 20,404 entries). Data were filtered in Perseus [28] and submitted for statistical analysis using PolyStest [39]. Data quality control was performed in Complex Browser [40] and uploaded into Ingenuity Pathway Analyzer (Qiagen, Hilden, Germany) for further analysis. Reactome v.77 pathway analysis tool was used for biological process enrichment analysis [41]. The data is available at ProteomeXchange Consortium via the PRIDE depository [42] with the dataset identifier PXD029200. Lists of identified proteins (before and after filtering) can be found in Supplementary Tables S3 and S4, respectively.

## 3. Results

**Figure 1:**
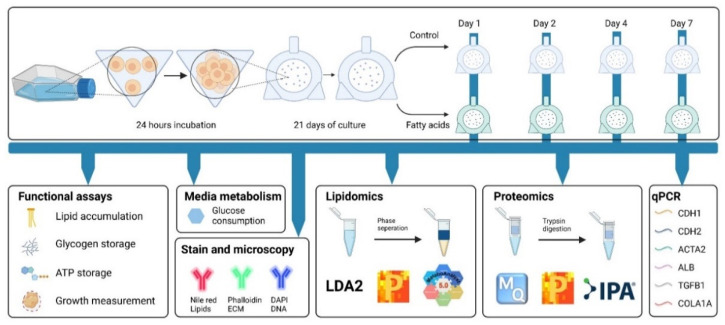
Experiment overview. Top panel—Creating and exposing HepG2/C3A spheroids to fatty acids (FA) (45 μM palmitic acid and 65 μM oleic acid conjugated to BSA). Cells are seeded into low attachment microwell plates. After 24 h, mini spheroids are transferred into rotary bioreactors and cultured for 21 days before exposure to FA. Samples are harvested after 0, 1, 2, 4 and 7 days of FA treatment. Bottom panel—measurements and tests performed in this study (see Section 2).

To understand the effects of elevated levels of free fatty acids on human hepatocytes, we created HepG2/C3A spheroids and, after 21 days of growth, exposed them to 45 μM palmitic acid and 65 μM oleic acid. Figure 1 (top panel) summarizes the creation and treatment scheme. Figure 1 (bottom panel) presents an overview of the assays performed and analytical methods applied throughout the project. Functional assays were performed to determine fatty acids’ effect on lipid accumulation, glycogen storage, ATP storage and spheroid growth. Fatty acid treatment’s effect on glucose uptake and the organization of the internal spheroid was investigated by glucose consumption assay, lipid staining and microscopy. Lipidomics and proteomics were employed to investigate quantitative changes in 637 lipid species and 4981 proteins. Affected signalling networks and biological processes were mapped and compared to the existing knowledge. Finally, the expression of selected genes involved in the identified networks was measured using quantitative PCR.

### 3.1. Spheroids Exposed to Palmitic and Oleic Acids Show Significantly Increased Levels of Intracellular Lipids

We characterized the metabolism of HepG2/C3A spheroids exposed to elevated fatty acid levels (45 μM palmitic and 65 μM oleic acid), focusing on traits characteristic of the early stages of fatty liver development. Spheroids grown for 21 days in rotary bioreactors show liver-like metabolic characteristics [43]. We expected a moderate increase in palmitic and oleic acid, corresponding to the difference observed in healthy versus NAFLD patients (post-meal) [19], which would only mildly affect the viability and function of these hepatocyte spheroids.

**Figure 2:**
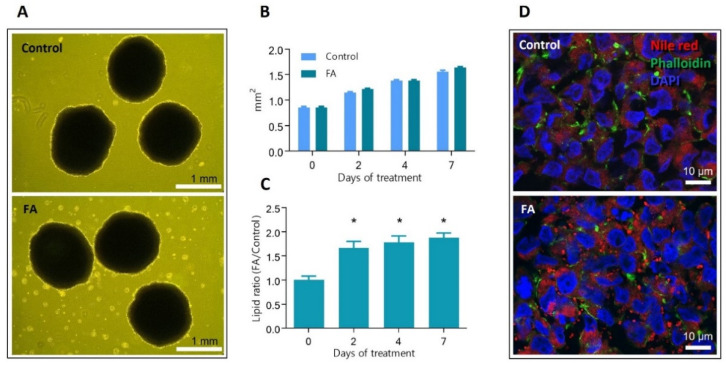
The effect of 45 μM palmitic acid and 65 μM oleic acid on spheroids growth and induced intracellular accumulation of lipids. FA—spheroids treated with 45 μM palmitic acid and 65 μM oleic acid, Control—spheroids in standard growth media. (**A**) 28 days old spheroids were maintained in growth media (upper panel) and after 7 days of FA supplementation (lower panel). Light microscopy images. (**B**) The growth of spheroids exposed to FA supplementation or Control media is determined by measuring spheroids’ planar surface. No statistically significant difference was observed between the Control and FA-treated spheroids at any time (*n* = 21, *p* < 0.05). (**C**) Relative intracellular lipid levels in FA-treated and non-treated spheroids at day 0 (before treatment), 2, 4 and 7. * indicate statistical significance between Control and FA spheroids at respective timepoint (*n* = 5, *p*-value < 0.05). (**D**) Fluorescent microscopy images of cryo-sectioned spheroids stained for DNA (blue), Lipids (red), and f-actin (green). Error bars show the standard error of the mean.

Figure 1 (top panel) depicts the course of the experiments. On day 21 of growth in rotary bioreactors, spheroids were supplemented with 45 μM palmitic acid and 65 μM oleic acid conjugated to bovine serum albumin, and the treatment was continued for 7 days. Figure 2A shows images of 28 days old spheroids, treated and untreated with palmitic and oleic acid (FA and Control, respectively). Measurements of spheroids’ size were carried out on exposure days zero, two, four, and seven, and no significant difference between treated and untreated spheroids were observed (Figure 2B). Spheroids were also tested for lipid content by AdipoRed assay (Figure 2C). The lipid levels were normalized to spheroids size and represented as a ratio against the Control. A significant increase in lipid accumulation was observed after two days of exposure and continued until the end of the experiment. The presence of intracellular lipid droplets was confirmed by fluorescent microscopy. Figure 2D shows representative images of cryosectioned spheroids stained for lipids—red, f-actin—green, and DNA—blue. A higher number of lipid droplets was observed inside the hepatocytes exposed to palmitic and oleic acids than in the Control, where only a few were present. These results show that supplementing culture media with 45 μM palmitic acid and 65 μM oleic acid induces rapid intracellular accumulation of fatty acids but does not affect HepG2/C3A spheroids growth.

### 3.2. Hepatocytes in Spheroids Remain Functional despite Slight Changes in ATP Production and Glycogen Storage Management

To understand the extent to which the hepatocytes were affected by the palmitic and oleic acid treatment, we measured the level of ATP in the cells, glucose uptake from media, and the cells’ ability to store glycogen and produce albumin. The ATP levels in Control spheroids were constant up to day four; on day seven, the ATP levels decreased slightly. That is probably related to the overall increased size of the spheroids. FA treatment of spheroids resulted in a small but significant increase in ATP levels on day 2 (18%), day 4 (13%), and day 7 (31%).

**Figure 3:**
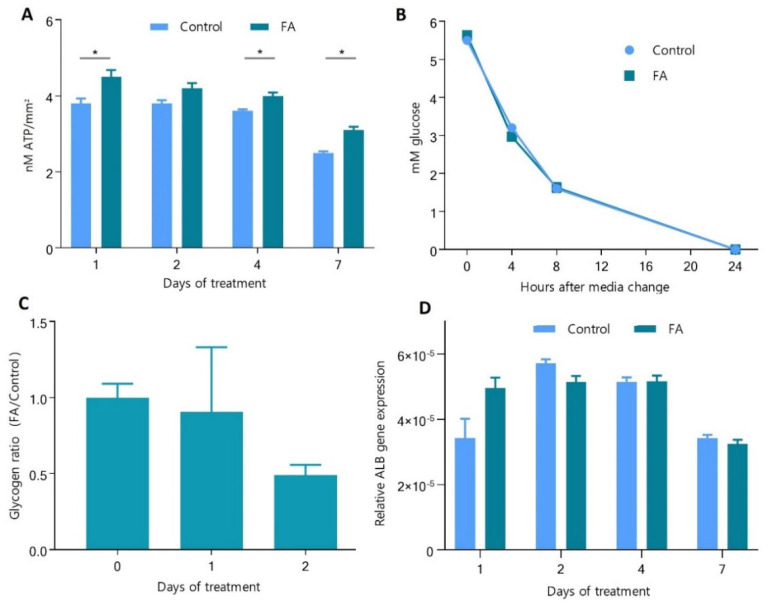
The effect of palmitic and oleic acids on hepatocyte function. FA-spheroids treated with 45 μM palmitic acid and 65 μM oleic acid, Control-spheroids in standard growth media. (**A**) ATP level in spheroids normalized by spheroids surface (*n* = 3). (**B**) Glucose consumption was measured in culture media from spheroids pre-exposed to FA for 4 days. It was followed at 0, 4, 8, and 24 h after supplementation with a fresh cell culture media (*n* = 3). (**C**) Glycogen deposition ratio in 21-day-old spheroids after 0, 1, and 2 days of FA exposure normalized to spheroids size and a respective control (*n* = 3). (**D**) Albumin gene expression after 1, 2, 4 and 7 days of treatment with FA (*n* = 6). * statistically significant difference between Control and FA-treated spheroids at the indicated timepoint, Student’s *t*-test and Holm-Sidak post hoc test, *p* < 0.05. Error bars show the standard error of the mean.

Next, we investigated the ability of hepatocytes to absorb glucose after four days of FA exposure. The glucose in the cell culture media was measured at 0, 4, 8, and 24 h after the media change. Rapid glucose uptake was observed, and after 24 h, glucose levels in the media were below the detection levels (Figure 3B). The Control and FA-treated spheroids showed a similar pattern; no significant difference was observed in glucose uptake.

Hepatocytes store excess energy in the form of glycogen, which in vivo is typically synthesized after a meal. In our model, glycogen is synthesized rapidly after glucose is taken up from the freshly supplied cell culture media (feeding phase, here 0–24 h after media change) and used by the cells when glucose in the media is consumed (fasting phase, here 24–48 or 72 h) until fresh glucose is provided as part of the routine media change procedure (every two or three days) [18]. To evaluate the impact of FA treatment on energy homeostasis in this model, we measured glycogen content in spheroids exposed to fatty acids. Samples were collected on day 0 in fasting cells and days one and two, corresponding to 24 h and 48 h after the cells were supplied with fresh media with glucose and fatty acids (Figure 3C). Glycogen content was 1.11 and 2.05-fold less in spheroids exposed to fatty acids. The measurements showed that cells supplied with standard glucose levels (5.6 mM) accumulated glycogen. However, cells supplied with additional FA did not follow that trend, suggesting that the fatty acids slightly affect the energetic homeostasis of hepatocytes and their ability to store glycogen.

Albumin (ALB) is one of the most abundant proteins in the blood and is synthesized and secreted by hepatocytes. ALB gene expression changes indicate an altered function of hepatocytes. In our study, the qPCR analysis showed no significant differences between spheroids exposed to fatty acids and Control (Figure 3D), suggesting no or minimal effect on this essential function of hepatocytes.

### 3.3. Palmitic and Oleic Acids Treatment Induce Lipidome Changes Like Those Seen In Vivo

To understand the impact of palmitic and oleic acid on the lipid composition of hepatocytes, we have carried out a quantitative lipidomics analysis of lipids extracted from HepG2/C3A spheroids. Spheroids were treated for zero, two, four and seven days with and without PA and OA. We observed significant changes in cellular lipid content and composition similar to those in NAFLD patients [44,45,46,47,48].

**Figure 4:**
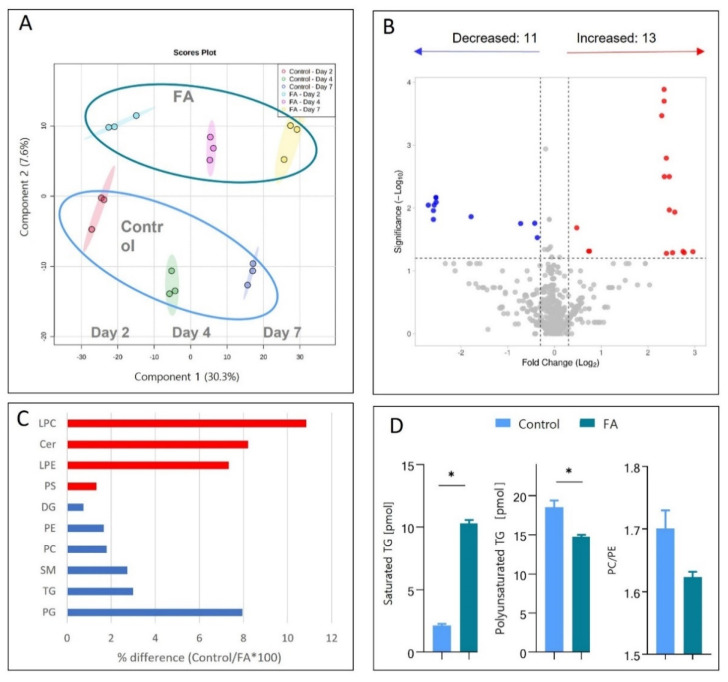
Lipidomics analysis of HepG2/C3A spheroids identifies lipids affected by palmitic and oleic acid treatment. FA-spheroids treated with 45 μM palmitic acid and 65 μM oleic acid, Control-spheroids in standard growth media. (**A**) The partial least square discriminant analysis of samples shows the grouping of Control (blue circle) and FA-treated samples (green circle). (**B**) Volcano plot of the lipid species altered in FA-treated spheroids (7 days) compared to control. The log2 fold change of FA vs. Control was plotted against the -log10 *p*-value evaluated by *t*-test. The significance threshold (dotted lines) was *p*-value < 0.05 and a fold change of 1.5. Dots in red and blue represent significantly decreased and increased species, respectively. (**C**) % difference after 7 days of FA exposure between control and treated spheroids. Red indicates a percentage increase in fatty acid-treated spheroids compared to control displayed in percentage. (**D**) From left, accumulation of saturated triglycerides (TGs) in FA-treated spheroids, decreased levels of polyunsaturated TGs, and decreased PC/PE ratio in FA-treated spheroids as compared to the control samples. * statistically significant difference according to Student’s *t*-test and Holm–Sidak post hoc test, *p* < 0.05, *n* = 3. Error bars show the standard error of the mean.

We have detected and quantified 1447 species of lipids using LDA2 software. The quantitative data were converted to absolute values using internal lipid standards. Lipid data was filtered, log2-transformed and compound names were assigned using the Metaboanalyst database to create a final list of 565 species (with 9.71% missing values) used for detailed statistical and biological analyses. Partial least squares discriminant analysis showed that the lipid composition of Control and treated spheroids changed over time (coloured points in Figure 4A (note that the coloured shadow behind the points indicates the variability of each sample group)). More importantly, the lipidomes of spheroids treated with fatty acids always differed from those of control spheroids, Figure 4A (green circle—FA-treated samples, blue circle—control samples).

Next, we wanted to understand whether the fatty acid treatment of spheroids induces lipidome changes similar to those observed in vivo. Based on previously reported studies [44], we selected ten classes of lipids that play an important role in the development of fatty liver or are observed to change in NAFLD patients; note that each class contains similar molecules of different structures or chain lengths. Figure 4C summarizes lipids from those classes measured on day 7 of FA treatment. The percentage difference in lipid class between Control and fatty acid-treated spheroids was calculated based on the summation of the total picomol of all molecular species identified in each class identified in Metaboanalyst for each sample. For example, 241.51 pmol of phosphoglycerols (PG) in the Control and 223.72 pmol PG in the (day 7) treated spheroids. 223.72 pmol/241.51 pmol × 100 = 92.63% equals a 7.37% decrease in fatty acid-treated spheroids compared to Control.

We observe an increase in the lysophosphatidylcholine (LPC) (+10.86%) [44], lysophosphatidylcholine (LPE) (+7.34%) [45] and ceramides (Cer) (+8.21%) [46] class. Phosphatidylserine (PS) (+1.33%) is not seen directly on biopsy comparing but seen upregulated in the serum of NAFLD patients compared to healthy controls [47]. These increases are similar to those observed in previous studies of NAFLD (as given in the references).

Phosphoethanolamines (PE) (−1.66%) [48], phosphatidylcholine (PC) (−1.80%) [49], sphingomyelins (SM) (−2.74%) [50] decrease, which also corresponds to studies of NAFLD patients (indicated by references). PG (−7.95%) indirectly relates to NAFLD since a decrease in PG has been previously related to mitochondrial dysfunction [51]. The changes observed in these studies thus echo changes reported in NAFLD literature related to NAFLD.

Triacylglycerol (TG) (−2.99%), and diacylglycerol (DG) (−0.75%) are seen to slightly decrease, which is not in agreement with the literature [45,52,53,54]. This disagreement is most likely due to the inability of Metaboanalyst and LDA to identify TG and DG lipid species in the lipidomics data.

These results are mere of an indicative nature since a statistically significant change only is seen when comparing the total composition of lipid classes of control spheroids to the total composition of lipid classes of the fatty acids-treated spheroids. That may be due to a high variation of some lipid species within each class. To investigate this, we looked at the individual lipid species and identified those that significantly changed upon FA treatment. We have used the *t*-test included in the Metaboanalyst 5.0 software and determined that FA treatment decreased levels of 11 and increased levels of 13 lipids as compared to the Control, out of the 570 different lipid molecule types quantitated Figure 4B (cut-off *p*-value < 0.05, fold change ± 1.2).

A detailed list of the significantly changing lipids is shown in Table 1. These individual lipid changes clearly show that not all lipids in the same class change in the same direction, indicating the significance of detailed lipidome analysis, Table 1**.** For example, only two phosphatidylcholine species are increased in FA-treated spheroids, and four species are decreased. Although not statistically significantly changing, Ceramides are also included in the table due to their involvement in inflammation and fatty liver development [52], Cer d28:2 shows a 6.83-fold upregulation which is statistically significant.

Another example is triglycerides. These are the primary lipid type accumulating in lipid droplets in fatty liver hepatocytes [48]. Interestingly, only two TG species increased in FA-treated spheroids, and seven species were significantly decreased Table 1. One significantly increased species is characterized by zero double bonds (TG (45:0)), a saturated TG. We compared the absolute concentrations of saturated versus unsaturated TG species (significantly changing) between the Control and FA-treated (7 days) spheroids, Figure 4D (left panel). That showed that there is 5.07-fold more saturated TG in FA-treated spheroids after 7 days of exposure, which agrees with the in vivo NAFLD studies [51].

One of the most frequent findings in in vivo NAFLD is a decrease in polyunsaturated fatty acids (PUFAs) [16]. We investigated if the polyunsaturated triacylglycerol (PUTG) expression also echoed what is seen in vivo. PUFAs are essential in shielding hepatocytes from lipotoxicity [53]. After FA treatment, the concentration of significantly regulated polyunsaturated triglycerides decreased significantly compared to control spheroids (Figure 4D (middle panel). PUTG content of FA-treated spheroids decreased 26% after just seven days of treatment, indicating that saturation of lipids mimics what is seen during NAFLD progression. A PC/PE ratio decrease was also observed in patients with NAFLD [54]. It is believed to be a result of the reorganization of the outer monolayer of cell membranes, causing decreased cell membrane integrity and heightened permeability towards cytokines, leading to activation of an immune response [55]. In our study, we calculated the ratio between the significantly changed PC/PE in the Control and FA-treated spheroids, Figure 4D (right panel)**.** Our results show a 5% decrease in the PC/PE ratio of significant species compared to the Control (as seen in vivo) after just seven days of treatment. That indicates that the spheroids can mimic PC/PE ratio changes in the early clinical onset of NAFLD.

In summary, the lipidomics analysis showed that fatty acid treatment induced lipidome changes similar to those observed in NAFLD patients, increasing ceramides, LPC and LPE and decreasing PC and PG. Deeper data interrogation revealed further NAFLD mimicry with increased saturated TGs, decreased polyunsaturated TGs and decreased PC/PE ratio, indicating a similar lipidomic response signature in FA-treated spheroids and NAFLD liver tissue.

### 3.4. Protein and Gene Expression Changes Pinpoint the Involvement of the TGF-β Pathway and the Transition of Hepatocytes towards a Mesenchymal Phenotype during Their Early Response to FA Overload

**Figure 5:**
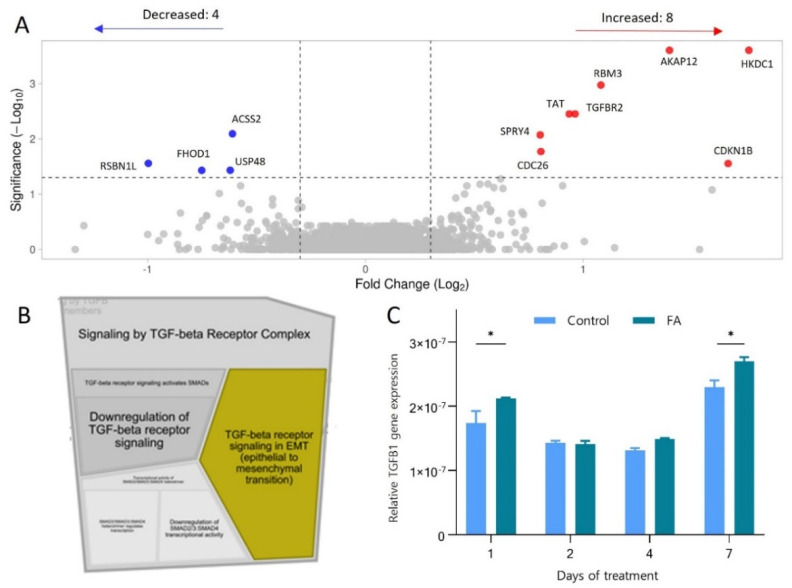
Proteomics reveals increased TGF-B signalling and epithelial to mesenchymal transition. FA-spheroids treated with 45 μM palmitic acid and 65 μM oleic acid, Control-spheroids in standard growth media. (**A**) Volcano plot depicting proteins regulated in FA-treated spheroids (7 days) compared to the Control. The log2 change of FA vs. Control is plotted against −log10 *q*-value calculated using PolyStest. The significance threshold (dotted lines) was set to *q* < 0.05 and a fold change of ± 1.2. Red dots-upregulated proteins, blue dots-downregulated proteins. (**B**) Fragment of the resulting pathways enrichment graph obtained using Reactome Pathway Database software [41]. Yellow indicates a statistically significant enriched category. (**C**) Relative TGF-B1 gene expression was measured by qPCR after one, two, four and seven days of treatment. * statistically significant difference according to Student’s *t*-test and Holm–Sidak post hoc test, *p* < 0.05, *n* = 3. Error bars show the standard error of the mean.

Spheroids were collected on days zero, two, four and seven during the fatty acid treatment and analysed using label-free quantitative proteomics and qPCR. The final dataset consisted of 4981 quantified proteins, with 6.8% missing values and a maximum coefficient of variance of 11.2%. Statistical evaluation carried out using PolySTest [39] revealed 12 significantly changed proteins (*n* = 3, *q* < 0.05, fold change 1.2 or greater) after 7 days of FA exposure. The results are presented in the form of a volcano plot in Figure 5A and listed in Table 2, including protein names, false discovery rates (FDR) and fold changes based on log2-transformed data.

Next, we performed gene ontology enrichment analysis using Reactome Pathway Database [41] using the proteins that were 1.2-fold (log2 transformed) upregulated or more (321 proteins). As a result, the TGF-β receptor signalling pathway for the epithelial to mesenchymal transition (EMT) was identified as the critical biological process involved in the early response of hepatocytes to increased fatty acid availability (Figure 5B). Indeed, among the upregulated protein, we identified TGFBR2 (fold change 1.95), a TGF-β receptor type-2 transmembrane serine/threonine kinase. Upon binding of TGF-β1 ligand to TGFBR2, receptor activation occurs by heterotetramer formation of TGFBR1 and TGFBR2 dimers, resulting in phosphorylation of TGFBR1 [56]. Unfortunately, TGFBR1 was not identified in the proteomics dataset. The upregulation of TGF-β signalling was confirmed by the significant increase of TGF-β1 mRNA after one and seven days of treatment (Figure 5C).

To confirm that TGF-β activates spheroids, we treated spheroids directly with TGF-β1 (2 ng/mL) for 14 days (adding fresh TGF-β1with each media change) and searched for downstream induction of mesenchymal phenotype markers. We searched for gene expression of N-cadherin (CDH2 as a marker for mesenchymal cell-type gene expression) and collagen type 1 alpha 1 chain (COL1A1 as a marker of myofibroblast function) by qPCR. The increased production of CDH2 and COL1A1 transcripts suggests that the hepatocytes transition towards a mesenchymal phenotype (Figure S1, Supplementary File S1).

**Figure 6:**
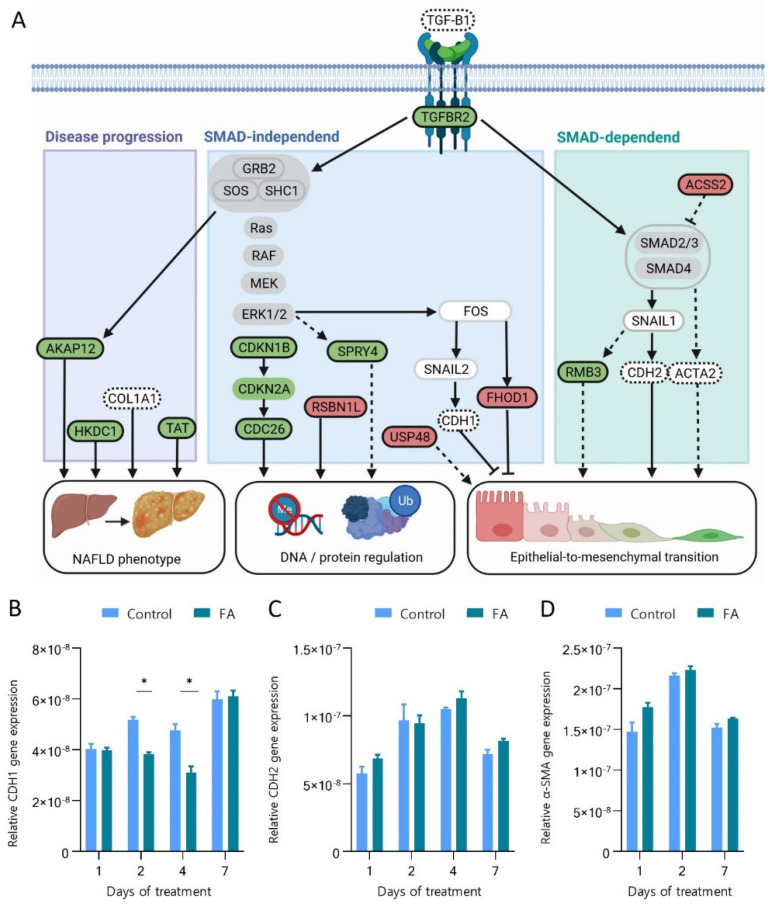
Signalling pathways and biological processes affected in spheroids treated with 45 μM palmitic acid and 65 μM oleic acid (FA) compared to the Control of standard growth media. (**A**) Summary of the dependencies between the significantly regulated proteins and their role in DNA and protein regulation, epithelial to mesenchymal transition and known markers of NAFLD phenotype in patients. The network was created using Ingenuity Pathway Analysis and BioRender tools. Circles indicate the following: Black-proteins significantly regulated FDR < 0.05; green-upregulated > 1.2-fold, red circles-downregulated > 1.2-fold, grey-not changing based on the proteomics data, white-not measured in this study, circled by dotted line-measured using qPCR. Full arrows indicate direct relation. Dotted arrows indicate indirect relations. Pointed arrows indicate activation. Blunt arrows indicate repression. Relative gene expression of (**B**) CDH1 (E-cadherin), (**C**) CDH2 (N-cadherin), and (**D**) a-SMA (alpha-smooth muscle actin) was measured by qPCR after 1, 2, 4 and 7 days of treatment. * statistically significant difference according to Student’s *t*-test and Holm–Sidak post hoc test, *p* < 0.05, *n* = 3. Error bars show the standard error of the mean.

To further understand the interplay between the up- and down-regulated proteins and their relation to epithelial-to-mesenchymal transition and other biological processes, data was analysed through Ingenuity Pathway Analyser [57]. We found that all the regulated proteins were connected to either the pathways leading to the activation of epithelial to mesenchymal transition, involved in the regulation of DNA transcription and protein degradation or were known proteins to be regulated similarly in NAFLD. Figure 6A summarises the molecular interactions of the 12 proteins found significantly changing after seven days of FA treatment mapped on the three main functional changes triggered by the activation of the TGFBR2 receptor. Proteins are described in detail in the following section, and which category they belong to is summarised in Table 2. The text in bold indicates proteins detected in this study to be significantly regulated.

We observe an increase in the abundance of **TGFBR2** and production of TGFB1 mRNA transcript, which indicates an activation of the TGF-β signalling pathway. TGF-β pathway downstream signalling is directed through either SMAD-dependent or SMAD-independent pathways. Our results show an increase of **ACTA2** (Alpha smooth muscle actin 2)**, CDH2** (N-Cadherin) **and RMB3** (RNA-binding proteome 3), which are activated by the SMAD-dependent transcription regulation. **ACTA2, CDH2 and RMB3** contribute to increased cell mobility, which is characteristic of the mesenchymal phenotype. **RBM3** (2.10-fold increased) defends against cell death caused by ER stress, hypoxia/ischemia, and other metabolic and disease-associated stressors [58]. Pilotte et al. found that using cell lines with increased RBM3, directional cell migration in a mesenchymal-like manner could be stimulated [59].

Additionally, we observed down-regulation of **ACSS2,** which further supports the activation of SMAD-dependent signalling. **ACSS2** (acetyl-CoA short-chain synthetase family member 2) (fold change −1.53) converts acetate to acetyl-CoA, which subsequently can be utilized in fatty acid synthesis [60]. Since there are excess fatty acids following treatment, substrates for fatty acid synthesis are unnecessary. Therefore, **ACSS2** is downregulated. That leads to a decreased production of acetyl-CoA and a decreased acetylation and activation of SMAD7, an inhibitor of SMAD2 phosphorylation [61]. Phosphorylation of SMAD2 is a necessary step in SMAD-dependent TGF-β1 signalling transduction leading to EMT. Decreased **ACSS2** thereby contributes to the downstream progression of transition from an epithelial to mesenchymal-like phenotype.

The TGF-β pathway also signals through a SMAD-independent route through the Ras/RAF/MEK/ERK pathway. TGF-β1 binding leads to TGFBR2, leading to a heteromeric complex of TGFBR1/TGFBR2 and phosphorylation of TGFBR1 by TGFBR2 [62]. This leads to phosphorylation of SHC1, causing the recruitment of GRB2 and SOS, causing further activation of Ras, resulting in ERK activation [63], which leads to further progress towards a mesenchymal phenotype. Activated ERK results in the activation of FOS and **CDKN1B,** and **SPRY4**. The activation of FOS leads to a decrease in **FHOD1** (FH1/FH2 domain-containing protein 1) and **CDH1** (E-Cadherin) through SNAIL2. A reduction of **FHOD1** and **CDH1** leads to a less epithelial phenotype. They are both proteins involved in the cell-to-cell connection of epithelial cells, which plays a crucial role in epithelial cell morphology. 

Specifically, **FHOD1** (fold change −1.69) impairs spreading, the synchronized application of adhesive force and adhesion maturing [64]. **FHOD1** is crucial in creating adherence junctions and enabling the connection between epithelial cells [65]. **CDH1** is the gene encoding for E-cadherin, also involved in cell-to-cell adhesion. It is used as a marker of epithelial cells and will be discussed in the section below [66].

Activated ERK signalling also results in increased activation of **CDKN1B**, which likewise is related to the progression of a mesenchymal phenotype (3.17-fold). **CDKN1B** encodes p27, a cyclin-dependent kinase inhibitor, mediating cell cycle inhibition and regulating normal cell cycle progression [67]. **CDKN1B** is also a transcriptional regulator, driving downstream activation of transcription factors of EMT [68].

Extracellular signal-regulated kinases (ERKs) activate **SPRY4** (Protein sprouty homolog 4) (1,75-fold increased) which is a part of the sprouty (SPRY) protein group that plays a crucial role in regulating proliferation, differentiation, migration and survival by inhibition of receptor tyrosine kinase-mediated extracellular signal-regulated kinase pathway [69]. Studies have shown signalling transduction from TGF-β1 via MAPK/ERK to SPRY4 using knockout mice [70]. Other SPRYs have been connected to EMT where SPRY2 upregulation promotes EMT [71] or SPRY1, which regulates change in morphogenesis and ECM, which is essential for EMT [71]. SPRY4’s role in EMT is, however, still unclear.

EMT is not only induced in SMAD-independent regulation via GRB2/SOS/SCH1 complex regulation of the Ras/RAF/MEK/ERK pathway. GRB2/SOS/SCH1 complex activation also results in the downregulation of USP48 (−1.53-fold change). **USP48** (Ubiquitin carboxyl-terminal hydrolase 48) hydrolyse and remove ubiquitin from proteins, determining the proteins’ destiny in downstream cell processes [72]. During EMT, the ubiquitin-proteasome system modulates vital proteins to acquire the mesenchymal phenotype essential for EMT [73]. USP48 is important in EMT since it stabilizes TRAF2 and thereby reduces E-cadherin mediated adherence junctions, leading to loss of an epithelial marker and thereby further gain of mesenchymal phenotype [74].

To document hepatocytes’ transition towards the mesenchymal phenotype, we measured the transcription of **CDH1, CDH2** and **ACTA2 genes** involved in the TGFBR2 signalling (Figure 6A). **CDH1** and **CHD2** encode calcium-dependent cell-cell adhesion glycoproteins called Cadherin-1 and 2. Cadherin-1 forms adherence junctions between epithelial cells, suppressing tumour invasion and metastasis [75]. The downregulation or loss of Cadherin-1 is a critical molecular feature of EMT and is associated with an increase in Cadherin-2 levels, the so-called “cadherin switch” [76]. The upregulation of Cadherin-2 induces increased motility, invasion, and metastasis [77]. **ACTA2** encodes alpha-smooth muscle actin (α-SMA), which is an established marker for the detection of myofibroblast-like cells (a mesenchymal cell type) [78]. Thus, its upregulation in hepatocytes indicates its progression towards the mesenchymal phenotype. Figure 6B shows a significant transient decrease in CDH1 gene expression after two and four days of treatment by −1.35 and −1.53-fold, respectively, compared to the corresponding Control. That could indicate a possible loss of epithelial junction structures in the hepatocytes. Days one and seven of exposure did not show a significant change. The transcription of CDH2 and ACTA2 showed no significant change after FA treatment, Figure 6C,D.

In summary, the exposure of human hepatocytes to increased levels of fatty acids leads to proteins and mRNA transcription changes associated with a mesenchymal phenotype.

### 3.5. Fatty Acids Increase Protein Ubiquitination and Degradation and Affect the Regulation of Chromatin Structure

Two proteins, **CDC26** (fold change 1.74) and **USP48** (fold change −1.53), involved in the ubiquitination of proteins and cell cycle progression, were affected by FA treatment. Interestingly **CDC26** is a ubiquitin ligase [79], and **USP48** is a ubiquitin-specific peptidase [80]. Thus, the two proteins have a synergistic regulatory effect. Their opposing direction of the regulation suggests a strong regulation towards increased protein ubiquitination and degradation, promoting cell cycle progression and proliferation. Both proteins have previously been associated with protein complexes involved in motility, EMT, metastasis or cancer progression [72,81,82,83].

**RSBN1L** (fold change −1.99) is a lysine-specific demethylase, and its function has not been studied so far. Its function has been determined by similarity [84], and it is most likely responsible for the demethylation of lysine residues on histone proteins, thus playing a role in regulating chromatin structure and gene expression. Histone tail methylation may result in gene silencing or activation, depending on the location of the amino acid substrate for methylation and the valency, mono-, di-, or tri-methylation. Therefore, it is difficult to predict the effect of the reduced levels of **RSBN1L**.

### 3.6. Hepatic 3D Spheroids Show Similar Protein Regulation as Observed in NAFLD

Three proteins, **AKAP12**, **TAT** and **HKDC1,** identified as upregulated in this study, are also known to be upregulated in NAFLD patients [85,86,87].

**AKAP12** (fold change 2.58) is an A-kinase anchor protein 12 (also known as gravin) that mediates subcellular anchoring of protein kinase A (PKA) and proteins kinase C (PKC) to the plasma membrane. **AKAP12** plays a significant role in regulating cellular adhesion through its Control of cytoskeleton architecture and cell migration. It also acts as a tumour suppressor, regulating cell-cycle progression [88]. Ramani and colleagues investigated the **AKAP12** role related to liver injury and found that **AKAP12** mRNA expression (quantified by qPCR) of human liver biopsies increased upon liver injury [85].

**TAT** (fold change 1.93) is a tyrosine aminotransferase involved in the degradation of tyrosine, a process that mainly takes place in the liver [89] and produces intermediates or precursors for gluconeogenesis and ketogenesis. The exact role of **TAT** and the consequence of its upregulation in the development of NAFLD is not fully understood. However, using metabolomics, Jin et al. have shown that tyrosine metabolism is the most dysregulated amino acid pathway in adolescent NAFLD patients and that plasma tyrosine levels are positively associated with the severity of steatosis [86].

**HKDC1** (fold change 3.72) is a hexokinase enzyme that catalyses hexose phosphorylation to hexose 6-phosphate, although its activity is low compared to other hexokinases [87]. Its overexpression in hepatocytes affects metabolism, leading to reduced glycolytic capacity, intensification of mitochondrial respiration and reduction in glucose oxidation, leading to increased ROS production and cell damage [87]. **HKDC1** expression was also elevated in human patients with advanced stages of NAFLD, and similar observations were made in mice on a high-fat diet causing high levels of liver inflammation and fibrosis [88].

Our proteomics analysis showed few significantly regulated proteins. Most of them were involved in or affected by TGF-β signalling. Some proteins were also involved in DNA and protein regulation, induction of a NAFLD-like phenotype and progression towards an epithelial-to-mesenchymal transition.

The 3D cell culture model employed in this study has enabled the culture of a liver model that responds to physiologically relevant levels of fatty acid exposure, reflecting changes in NAFLD patients in vivo. Thus, this in vitro model enabled in-depth proteomics and lipidomics analysis of early NAFLD development, revealing proteome and lipidome signatures similar to those observed in vivo in NAFLD patients. Furthermore, evidence-based proteomics analysis suggests that EMT has increased relevance in the early onset of NAFLD and disease development.

## 4. Discussion

This study aimed to map the molecular pathways affected by physiologically relevant levels of fatty acids in human hepatocytes. Such analysis is difficult, if not impossible in vivo. However, by using the human hepatocytes-based 3D cell culture model, we have better understood how fatty acids affect hepatocytes. We investigated functional, lipidome and proteome changes in 21-day-old liver spheroids during seven days of fatty acid (65 μM OA and 45 μM PA conjugated to BSA) treatment.

A hallmark of NAFLD is the accumulation of lipids in the liver [1]. This change was observed in the treated spheroids with spectroscopy and fluorescent microscopy. Lipid accumulation has been found in other 3D in vitro models with similar fatty acid treatment [22,89]. In contrast to this study, they applied an additional risk factor by using hyperglycaemic glucose concentrations (five times higher than our study). The analysis performed during this study used, to our knowledge, the lowest metabolic load resulting in lipid accumulation in a dynamic 3D in vitro model concerning NAFLD.

Upon fatty acid exposure, we did not observe any change in albumin expression. Albumin is a crucial protein produced by hepatocytes and used to measure cell functionality, especially in the 3D liver in vitro models [90]. Our result indicates no change in hepatocyte function when exposed to fatty acids, despite albumin’s role in binding fatty acids [91]. Kozyra et al., similarly, found no significant difference in albumin levels in the 3D liver model when treating it with 80 μM palmitic and 80 μM oleic acids [22].

The ATP content of FA-treated spheroids was higher than that of Control. While ATP can be used to measure viability, the increase in ATP levels is likely caused by the increase in β-oxidation rate. As free fatty acids are direct substrates for this process, degradation of non-accumulated fatty acids would lead to ATP production. Such an increase in ATP levels was also seen by Feaver et al. [23].

A 50% decrease in glycogen storage was observed after two days of fatty acid exposure. Similar changes have been seen after palmitic acid treatment in a rodent model resulting in a 51% decreased insulin-stimulated glycogen synthesis [92]. That indicates that PA is not only stored in lipid droplets but also causes changes in insulin signalling and subsequent glycogen storage. Palmitic acid induces insulin resistance in HEPG2 cells through enhanced ubiquitination and proteasomal degradation of key insulin signalling molecules [93]. That correlates with the increased ubiquitination observed in our proteomics data. Insulin resistance is a risk factor for NAFLD, and type 2 diabetes [5]. However, the exact molecular interplay is complex and still not fully delineated.

Our results show that the lipidome of hepatic spheroids changes significantly upon exposure to PA and OA. We have demonstrated that eight of nine NAFLD-relevant lipid classes change similarly as observed in vivo. Lipidomics allowed the analysis of cellular lipids at the level of individual lipid species and identified lipids that mimic the changes seen in NAFLD. Concentrations of the significantly changing species of saturated fatty acids were higher in the FA-treated spheroids. Such an increase in saturated fatty acid levels is also observed in vivo and leads to endoplasmatic stress, ROS production, apoptosis and inflammation [94].

The FA-treated spheroids have significantly lower levels of polyunsaturated triacylglycerols (PUTGs), the most commonly observed change between healthy and NAFLD patients. The increase in saturated fatty acids and the decrease of PUTGs lead to an overall rise in lipid saturation, which could be particularly interesting for further investigation during the early-onset NAFLD. Other 3D models of NAFLD have not observed this difference [24]. This could be attributed to the relatively low levels of fatty acids used in this study that allowed us to focus our analysis on identifying the early changes occurring during the development of the NAFLD phenotype.

Some changes in lipid composition of hepatocytes that were not widely recognized as relevant in NAFLD are, for example, the depletion of PG. PG is a precursor for cardiolipin synthesis, a phospholipid necessary for mitochondrial function [95]. A lack of PG could lead to decreased cardiolipin levels and mitochondrial dysfunction [95] which is one of the critical molecular events occurring in NAFLD. It disturbs hepatic lipid homeostasis and induces ROS production, cytokine release, and lipid peroxidation, leading to cell death [96]. Based on these data, one could postulate that molecular changes in the mitochondria of hepatocytes cause NAFLD. That is where intervention would be beneficial with drugs or increasing the intake of protective lipids [97]. It could indicate that mitochondria dysfunction is key to progressing from steatosis to NASH.

TG and DG accumulate in NAFLD. That was also replicated in our system by lipid accumulation using spectroscopy (AdipoRed) and confocal microscopy (NileRed), but not confirmed by lipidomics analysis, TG (−1.47%) and DG (−0.37%). We speculate that this is related to lipidomics sample preparation, MS analysis, filtration, or identification of lipid species. Upon further investigation after filtering and identification, 54% of species identified in the PC lipid class remain, whereas 35% and 46% of species remain for DG and TGs, respectively. Therefore, there is a substantial difference in glycerolipid (DG and TG) compared to phospholipid identifications (e.g., PE, PC, PS). This could be attributed to the complexities of aliphatic moieties and the position of the glycerol backbone. DGs and TG species identification and quantitation are difficult, even with today’s lipidomics advancement [98]. This lipidomics data shows the importance of developing better lipid species identification methods.

We identified 12 significantly regulated proteins in response to the fatty acid treatment. Of specific interest was HKDC1, which had a 3.7-fold increase in FA-treated spheroids. The hepatic version of HKDC1 is highly involved in metabolism [87]. A knockout mouse model has shown its relevance in maintaining whole-body glucose homeostasis [99]. The same study showed decreased energy storage levels, which offers some mechanistic understanding of the observed lack of glycogen storage. Other studies showed a connection between HKDC1 and mitochondrial respiration [87], which could be linked to increased ATP production. That study further showed that increased expression of HKDC1 caused mitochondrial dysfunction and even saw an increased expression of this protein in patients with NAFLD [87]. That underlines HKDC1s relevance in NAFLD and the need for further research into this protein’s role and its potential as a drug target.

We found an enrichment of proteins involved in TGF-β signalling and investigated the expression of TGF-β1 using transcriptomics. After seven days of fatty acid exposure, we found increased expression of TGF-β1. A previous study aiming to find non-invasive biomarkers of NAFLD using proteomics analysis found a strong association of TGF-β1 with NAFLD [100]. These findings suggest that an increase in TGF-β-signalling is connected to the progression of NAFLD. Other studies have also observed increased TGF-β signalling in NAFLD patients [101]. This pathway activates a myriad of downstream processes. The wound-healing response, cell survival, and differentiation are particularly interesting for this study [102]. TGF-β is also an inducer of stellate cell activation of co-cultures of hepatocytes and stellate cells. It caused a fibrogenic phenotype with a significant increase in collagen production [103]. After seven days of TGF-β treatment, our model also acquired a more fibrogenic phenotype. We found a significant increase in mRNA encoding COL1A1 and CDH2, indicating a cell’s transition towards a mesenchymal phenotype. These findings suggest that hepatocytes retain some amount of plasticity and can undergo at least partial epithelial-to-mesenchymal transition and develop towards a mesenchymal phenotype.

EMT is a reversible process characterized by a gradual loss of cobble-stone epithelial morphology and a gain of spindle-shaped appearance and mesenchymal function [104]. TGF-β is one of the most potent inducers of EMT in physiological and pathological contexts [105]. Activation of the TGF-β pathway upon fatty acid treatment in a 3D NAFLD model has previously been reported by both Mukherjee [106] and Hurrell [107]. TGF-β is used in various models to activate stellate cells, mainly to induce a fibrotic phenotype in a co-culture model of hepatocytes, stellate cells, and other cells [103,108]. However, it has not been proven that stellate cells are the only source of myofibroblasts. By culturing hepatocytes, we were able to investigate if they could undergo EMT. EMT has been linked to fibrosis in the kidney, lung, intestine, and other organs [109]. It is widely accepted that renal epithelial cells give rise to renal myofibroblasts [110]. However, EMT in liver fibrosis is still controversial and not part of the consensus mechanism in NAFLD pathophysiology [111]. Numerous studies have proved that hepatocytes can obtain a fibroblast-like phenotype [110,112,113,114]. For example, Dooley and colleagues show in liver biopsies that collagen and transferrin were co-expressed, demonstrating the likely incidence of EMT [110]. To further link TGF-β signalling and EMT, they inhibited TGF-β signalling in hepatocytes and saw a reduced fibrogenic response.

Yu and colleagues [115] have reviewed and discussed the current evidence for the involvement of EMT in liver fibrosis. Several studies suggested that hepatocytes could acquire a fibroblastic phenotype through EMT in liver fibrosis, as discussed in [115]. However, Taura et al. [116], using transgenic mice and lineage tracing, found that hepatocytes failed to express mesenchymal markers including FSP-1, α-SMA, and vimentin and were not the origin of type I collagen-producing cells in the liver. That work challenges the existence of hepatocyte EMT, but it is still too early to exclude its role in liver fibrosis and NAFLD disease progression. Delineating the role of EMT in NAFLD could help prevent fibrosis from occurring, which is a critical factor influencing the survival of NASH patients [117]. Other 3D fibrotic cell model studies have shown attenuation of fibrogenesis upon anti-fibrotic drug administration [108], indicating that preventing EMT also leads to a decrease in fibrosis.

It is conceivable that the origin of the contradictory results reported by different groups studying the involvement of ETM in the development of NAFLD may stem from the use of different experimental models. In our study, we chose to create spheroids based on the HEPG2/C3A cell line, which is a subclone of the HEPG2 hepatoma cell line. HEPG2/C3A were isolated based on their increased hepatocyte-like phenotype, inability to form tumours in nude mice and ability to proliferate and survive in physiological levels of glucose (5.6 mM) [118]. We have selected to culture the HEPG2/C3A cells in 3D because it has been shown to improve their metabolic mimicry of in vivo hepatocytes [18]. This choice allowed us to investigate the changes during relatively long-term exposure of hepatocytes, avoid problems with material availability and optimise the cost efficiency of the experiments [119]. Primary hepatocytes or stem cells were not chosen for this study due to their limited viability in cultures [120] and high donor-dependent variability, and rapid de-differentiation in vitro, as well as the immature phenotype of hepatocytes derived from stem cells [121].

One could argue that the malignant origin of the HEPG2/C3A cell line could contribute to the observation of EMT upon fatty acids overload of hepatocytes. EMT is one of the hallmarks of cancer development and one of the molecular changes underlying the appearance of a migratory phenotype and metastasis [122]. It is necessary to evaluate whether EMT caused by fatty acid exposure is promoted by the origin of the immortalized cell line. EMT was not previously studied in 3D NASH models based on primary cells [24,89].

We show that this model recapitulates in vivo disease progression as indicated by lipid and proteomic changes. It has been previously shown that the HEPG2/C3A spheroids are a good model for predicting LD50 values of drugs [17,123] and can be grown for up to 302 days allowing for studies mimicking the chronic disease development in humans [124]. Thus, in the future, it can be used to study the efficacy and toxicity of new drugs and interventions ameliorating the effects of increased levels of circulating fatty acids and a high-fat diet and can aid in the discovery and testing of novel drugs for the treatment and prevention of NAFLD.

## 5. Conclusions

In this study, we describe a model for early steatosis in NAFLD based on the 3D culture of human hepatic carcinoma cells exposed for seven days to physiological levels of fatty acids (65 μM oleic acid and 45 μM palmitic acid). We showed the NAFLD hallmark of lipid accumulation in our model and other changes in the lipidome similar to that seen in vivo. Especially interesting were the increased saturated fatty acids, the depletion of PUTGs and the increased PC/PE ratio. We also saw increased TGF-β1 signalling and its downstream effects, including molecular changes indicating development towards a mesenchymal-like phenotype of hepatocytes.

## Figures and Tables

**Table 1:** Lipid species were significantly regulated in spheroids treated with 45 μM palmitic acid and 65 μM oleic acid compared to control grown in standard growth media. Statistical analysis was performed using a Student’s *t*-test built in the Metaboanalyst v 5.0 software, *p* < 0.05, *n* = 3. CE (22:5) and Cer (30:0) though not statistically different, were included due to their importance in NAFLD development.

Class	Name	Fold Change	*p*-Value
Ceramides (Cer)	CE (22:5)	1.57	0.27
	Cer (d28:2)	6.83	0.05
	Cer (30:0)	1.46	0.34
Diacylglycerol (DG)	DG (42:9)	5.48	0.01
	DG (36:6)	−1.11	0.05
Lysophosphatidylcholine (LPC)	LPC (24:2)	2.6	0.00
Lysophosphatidylethanolamine (LPE)	LPE (13:0)	1.68	0.05
	LPE (26:2)	1.09	0.02
Phosphatidylcholine (PC)	PC (46:8)	5.94	0.01
	PC (42:12)	5.48	0.00
	PC (31:2)	−1.08	0.02
	PC (44:11)	−1.08	0.04
	PC (33:5)	−1.14	0.00
	PC (16:0/18:2)	−5.79	0.01
Glycerophosphoethanolamines (PE)	PE (43:6)	5.09	0.00
	PE (16:0/18:2)	−1.08	0.02
	PE (37:2)	−5.79	0.01
Phosphoglycerols (PG)	PG (28:2)	5.24	0.00
	PG (44:2)	−3.09	0.01
	PG (47:4)	−5.94	0.01
Phosphatidylserine (PS)	PS (37:4)	7.77	0.05
	PS (35:1)	−6.48	0.03
Triacylglycerol (TG)	TG (18:0/18:2/20:0)	5.25	0.00
	TG (45:0)	5.07	0.00
	TG (47:2)	−1.05	0.04
	TG (43:1)	−1.16	0.05
	TG (43:2)	−1.29	0.03
	TG (47:4)	−1.34	0.02
	TG (55:0)	−1.65	0.02
	TG (42:4)	−6.01	0.01
	TG (50:7)	−6.01	0.02

**Table 2:** Proteins significantly regulated in spheroids treated with 45 μM palmitic acid and 65 μM oleic acid compared to control grown in standard growth media after 7 days of treatment. Statistical analysis was performed using PolyStest (*p* < 0.05, *n* = 3).

Name	Gene Name	Fold Change	*Q*-Value
DNA and protein regulation			
Anaphase-promoting complex subunit CDC26	CDC26	1.74	0.0169
Ubiquitin carboxyl-terminal hydrolase 48	USP48	−1.53	0.0369
Lysine-specific demethylase RSBN1L	RSBN1L	−1.99	0.0277
NAFLD phenotype			
Hexokinase HKDC1	HKDC1	3.72	0.0002
A-kinase anchor protein 12	AKAP12	2.58	0.0002
Tyrosine aminotransferase	TAT	1.93	0.0035
EMT transition			
Cyclin-dependent kinase inhibitor 1B	CDKN1B	3.17	0.0279
RNA-binding protein 3	RBM3	2.10	0.0011
TGF-beta receptor type-2	TGFBR2	1.95	0.0035
Protein sprouty homolog 4	SPRY4	1.75	0.0084
Acetyl-coenzyme A synthetase	ACSS2	−1.53	0.0080
FH1/FH2 domain-containing protein 1	FHOD1	−1.69	0.0370

## Data Availability

Lipidomics and proteomics datasets generated and analysed in this study are publicly available. The data can be found at https://www.ebi.ac.uk/metabolights under accession number MTBLS3580 and at https://www.ebi.ac.uk/pride under accession number PXD029200.

## Supplementary Materials

The following supporting information can be downloaded at: https://www.mdpi.com/article/10.3390/cells11203216/s1. Supplementary File S1: List of internal standards used for lipidomics analysis, Lipidomics mass spectrometry settings, Proteomics search engine settings, and supplementary Figure S1. Supplementary data file containing: Table S1: All identified lipids with their absolute concentration in pmol; Table S2: Filtered absolute values of lipid used for biological analysis; Table S3: All identified proteins expressed; Table S4: Filtered expressed proteins used for biological analysis.
