# Supplementary material for: Mapping Proteome and Lipidome Changes in Early-Onset Non-Alcoholic Fatty Liver Disease Using Hepatic 3D Spheroids
Source: Cells. 2022 Oct 13;11(20):3216. doi: 10.3390/cells11203216 (PMC9600727; doi:10.3390/cells11203216)
Supplement: Supplementary file 1 [file cells-11-03216-s001.zip › Supplementary File S1.pdf]

Supplementary File S1

Mapping proteome and lipidome changes in early-onset non-alcoholic fatty liver disease using hepatic 3D spheroids

Helle Sedighi Frandsen[1,2], Joel Mario Vej-Nielsen[1,2], Lauren Elisabeth Smith[1], Lang Sun[4,5], Karoline Lindgaard Mikkelsen[1,3], Annemette Præstegaard Thulesen[1], Christina Erika Hagesen[1], Fuquan Yang[4,5], Adelina Rogowska-Wrzesinska[1]

[1] Department of Biochemistry and Molecular Biology, University of Southern Denmark, Odense, Denmark

[2] Sino-Danish College (SDC), University of Chinese Academy of Sciences, Beijing, China

[3] CelVivo ApS, Odense, Denmark

[4] Key Laboratory of Protein and Peptide Pharmaceuticals & Laboratory of Proteomics, Institute of Biophysics, Chinese Academy of Sciences, Beijing, China

[5] University of Chinese Academy of Sciences, Beijing, China

List of internal standards used for lipidomic analysis

Class	Supplier	Catalogue number	Concentration in std mix	Name	µg/mL	Exact mass	Formula weight (g/mol)	pmol/L
Cer	AVANTI	860517P-25mg	4 µg/mL	C17 Ceramide (d18:1/17:0)	4	551.5280	551.9270	7.247335
DG	AVANTI	800854P-1mg	12 µg/mL	1,3-17:0 D5 DG	12	601.5700	602.0100	19.93322
LPC	AVANTI	855676P-25mg	4 µg/mL	17:0 Lyso PC	4	509.6570	509.3480	7.853177
PE	AVANTI	860374P-25mg	4 µg/mL	16:0-d31-18:1 PE	4	748.7250	749.1870	5.339121
PG	AVANTI	860384P-10mg	4 µg/mL	16:0-d31-18:1 PG	4	801.7020	802.1800	4.986412
PS	AVANTI	860403P-25mg	4 µg/mL	16:0-d31-18:1 PS	4	814.6970	815.1790	4.906898
TG	AVANTI	860903P-1mg	12 µg/mL	17:0-17:1-17:0 D5 TG	12	851.7990	852.4150	14.07765

Lipidomics mass spectrometry settings

Dionex UltiMate 3000 nanoLC system had a flow of 250 µL/min with a gradient of 0min=10%B, 6min=60%B,18 min=100%B. A=MeOH CAN, H20(1:1:1) 5 mM NH4AC. B=IPA, MeOH=8. 25mMNH4AC. Injection volume was set to 2 µL. Lipids were analysed using a Triple Tof 5600 (AB Sciex) with the following settings: Vacuum gauge 3.1*10^-5 Torr, Source temp= 600C, Nominal 40 C in column oven. Lipids were analysed in positive ion mode with a vacuum gauge of 3.0. Time-of-flight (TOF) analysis was performed with the following settings: Number of cycles performed 1470, period cycle time 1300 ms, pulser frequency 16.633 kHz, accumulation time 250 ms and mass range from 100-1250. Tandem mass spectrometry was also performed on the same cycles in TOF-TOF mode with the following settings: Number of cycles 1470, positive polarity, period cycle time 1300 ms, independent data acquisition, pulser frequency 16.633 kHz, accumulation time 100 ms and mass range 50-1250.

Proteomics search engine settings

The equivalent of 1µg of peptide was taken for proteomic analysis. Peptides were captured on a commercial µ-precolumn (5µm, 5mm x 300 µm, 100 Å pore size, Acclaim PepMap 100 C18, Thermo Fisher Scientific, Bremen, Germany) before being separated using a home-packed column (1.9µm, 50cm x 100 µm, 120 Å pore size) of ReproSil-Pur C_{18} AQ beads (Dr Maisch GmbH, Ammerbuch-Entringen, Germany) heated to 50°C, at 8-35% buffer B (99.99% ACN, 0.01% FA) for 160 mins, at 550nL/min with a Dionex UltiMate 3000 nanoLC system (Thermo Fisher Scientific). Buffer A was 0.1% formic acid in water. Peptides separated online by reversed-phase LC were analysed by MS/MS using an Orbitrap Fusion Lumos Tribrid mass spectrometer (Thermo Fisher Scientific). A full MS scan across the mass range 350-1800 *m/z* was performed within the orbitrap with a resolution of 120,000 and an AGC target of 1×10^6 ions, with a maximum injection time (IT) of 50ms, a dynamic exclusion window of 40 sec and using Advanced Peak Detection. This was followed by fragmentation of selected ions with charge states of +2-6 by HCD within the ion trap. MS/MS scans were performed at a rapid ion trap scan rate, with a collision energy of 32%, a maximum IT of 35ms and an AGC target of 5×10^4. An isolation window of 1 *m/z* was used, with an isolation offset of 0.2 *m/z*; the total cycle time for the method was set to 2 sec.

Generated data were analysed using MaxQuant (v 1.6.2.10), with database searching performed using the in-built Andromeda search engine against the human UniProt Reference Proteome database (containing Swiss-Prot proteins including isoforms) (downloaded 25 February 2019, containing 20,404 entries). Data were searched with the following conditions: trypsin digestion with a maximum of 2 missed cleavages, a fixed modification of Carbamidomethylation (C), variable modifications of oxidation (M), Acetylation (Protein N-term) and Deamidation (NQ). Label-Free Quantitation was performed and the parameters "match between runs" and "second peptide search" were enabled and disabled, respectively; all other parameters remained as default.

Supplementary Figure S1

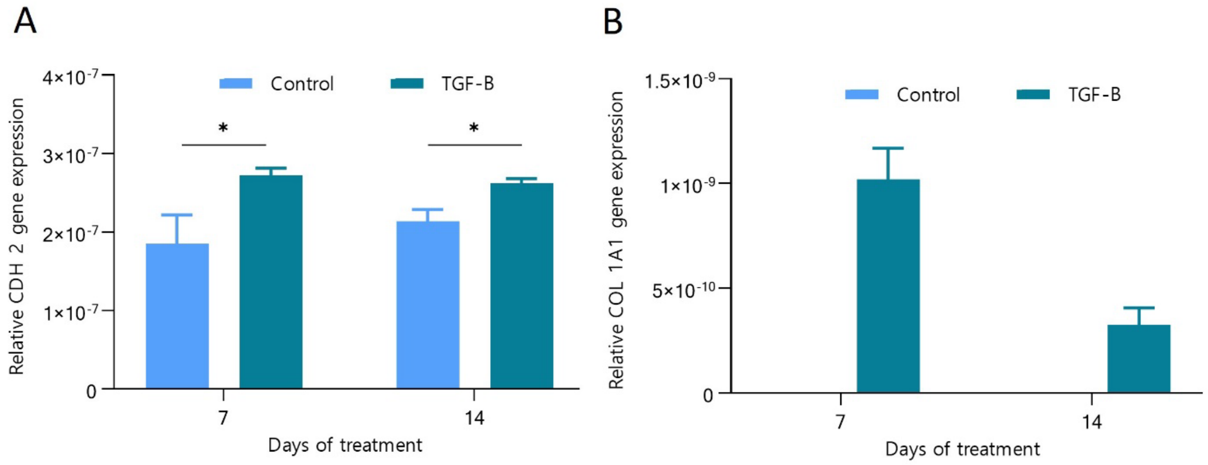

Figure S1. TGF-B treatment significantly increases mesenchymal marker and myofibroblast function. Relative CDH2(N-cadherin) gene expression (mesenchymal cell marker) was measured by qPCR after 7 and 14 days of treatment. (B) Relative COL1A1(collagen type 1 alpha 1 chain) and gene expression (myofibroblast function) were measured by qPCR after 7 and 14 days of treatment. Error bars show the standard error of the mean.
